# Keratinocyte necroptosis in inflammatory skin diseases and acute epidermal injury: mechanisms, evidence, and therapeutic implications

**DOI:** 10.3389/fimmu.2026.1967855

**Published:** 2026-10-02

**Authors:** Xu Liu, Di Qu, Jinyan Luo, Bo Chen, Xin Zhao, Zhengxu Fu, Jingdong Wu, Fan Xia, Yan Yan

**Affiliations:** 1 The First Clinical College, Liaoning University of Traditional Chinese Medicine, Shenyang, China; 2 Department of Rehabilitation, Children's Hospital of Shenyang City, Shenyang, China; 3 Chongqing Three Gorges Medical College, Chongqing, China; 4 Guangdong Provincial Key Laboratory of Physical Activity and Health Promotion, Guangzhou Sport University, Guangzhou, China; 5 Department of Infection Management Section, Shenyang First People's Hospital, Shenyang, China; 6 The First Clinical College, Shenyang Medical University, Shenyang, China; 7 Department of Endocrinology and Metabolic Diseases, Shenyang First People's Hospital, Shenyang, China

**Keywords:** epidermal injury, inflammatory skin diseases, keratinocyte, necroptosis, skin immunity

## Abstract

The skin functions as a barrier against external insults and as an active immune organ. Keratinocytes are central to both roles, maintaining epidermal integrity while coordinating cutaneous immune responses. Necroptosis is a lytic form of regulated cell death that can compromise the epidermal barrier and release damage-associated molecular patterns, thereby linking tissue injury to immune activation. Accumulating evidence implicates keratinocyte necroptosis in multiple inflammatory skin diseases and acute epidermal injuries. This mini review summarizes the molecular regulation of keratinocyte necroptosis, critically appraises evidence for its involvement across distinct pathological contexts, and evaluates therapeutic strategies and associated translational challenges. Mechanistic studies and preliminary translational evidence suggest that necroptosis-related signaling in keratinocytes may represent a therapeutic target. However, the effects of pathway modulation may vary across disease contexts and depend on infection status and the immune microenvironment. Defining the disease-specific contribution of keratinocyte necroptosis and the corresponding therapeutic windows will therefore be essential for evaluating the clinical feasibility of this strategy.

## 1 Introduction

The physical, chemical, and microbial barriers of the skin collectively form the body’s first line of defense against external insults and maintain local tissue homeostasis (1, 2). As the predominant cell type in the epidermis, keratinocytes not only form the physical barrier but also sense microorganisms, tissue damage, and environmental stress through pattern-recognition receptors. In response, they secrete cytokines, chemokines, and antimicrobial peptides and engage in bidirectional communication with immune cells, the skin microbiota, fibroblasts, and sensory neurons (3–5). When this regulatory network becomes dysregulated, keratinocytes can shift from maintaining tissue homeostasis to serving as key initiators and amplifiers of inflammation, thereby promoting the development and persistence of inflammatory skin diseases (6, 7).

Necroptosis is a lytic form of regulated cell death characterized by loss of plasma membrane integrity. In keratinocytes, it can cause cell loss and compromise the epidermal barrier, while the release of damage-associated molecular patterns may promote immune cell recruitment and amplify local inflammation. Keratinocyte necroptosis may therefore represent an important mechanistic link between barrier damage and immune activation (8, 9). However, evidence linking keratinocyte necroptosis to cutaneous inflammation remains fragmented, with substantial variation in mechanistic support and translational relevance.

Unlike previous reviews that broadly discuss necroptosis and related signaling pathways in cutaneous inflammation, this Mini Review adopts a keratinocyte-centered, evidence-stratified perspective. Rather than proposing a new pathway, it organizes dispersed findings across inflammatory skin diseases and acute epidermal injuries within a comparative framework. Specifically, it distinguishes altered pathway-protein abundance and pathway activation from mixed lineage kinase domain-like protein (MLKL)-dependent necroptotic execution, while separately considering the non-necroptotic functions of receptor-interacting protein kinases 1 and 3 (RIPK1 and RIPK3) and the context-dependent roles of MLKL. The evidence is critically appraised according to its source, cell-type specificity, mechanistic rigor, and translational relevance. This framework clarifies where keratinocyte necroptosis is mechanistically supported, where causal attribution remains uncertain, and which barriers constrain therapeutic translation.

## 2 Literature search and study selection

A structured literature search was conducted in PubMed and Web of Science from database inception to September 13, 2026, using terms related to necroptosis and cell-death signaling (e.g., “necroptosis,” “RIPK1,” “RIPK3,” “MLKL,” “ZBP1,” and “cell death”), keratinocytes and cutaneous tissues (e.g., “keratinocyte,” “skin,” and “epidermis”), and relevant disease or injury contexts (e.g., “psoriasis,” “interface dermatitis,” “atopic dermatitis,” “cutaneous infection,” “contact hypersensitivity,” and “acute epidermal injury”). Reference lists of relevant original articles and reviews were also screened. Detailed appraisal of acute epidermal injury was restricted to ultraviolet B (UVB)-induced injury and Stevens–Johnson syndrome/toxic epidermal necrolysis (SJS/TEN), for which sufficient direct keratinocyte-focused mechanistic or human-derived evidence was identified.

Eligible studies examined necroptosis-related signaling or MLKL-dependent cell death in keratinocytes or epidermal tissue using patient tissues, human-derived models, keratinocyte cultures, cell-type-specific or systemic genetic models, or pharmacological or clinical interventions. Peer-reviewed original studies were prioritized. Studies were excluded if they lacked direct relevance to keratinocytes or the cutaneous contexts addressed in this review, did not assess necroptosis-related signaling, provided insufficient mechanistic or methodological information, lacked accessible full text, or consisted of conference abstracts, editorials, or non-peer-reviewed reports. Reviews were used only for background and citation tracing. Consistent with its narrative design, this review provides a representative rather than exhaustive synthesis of the literature.

## 3 Molecular mechanisms of keratinocyte necroptosis

### 3.1 Canonical tumor necrosis factor receptor 1-dependent necroptosis signaling

Death receptors, pattern-recognition receptors, and nucleic acid sensors can initiate necroptosis under defined conditions, ultimately converging on the RIPK3–MLKL execution axis. However, the requirement for RIPK1 varies with the initiating stimulus and cellular context (10, 11). In canonical tumor necrosis factor-α (TNF-α) signaling, TNFR1 activation assembles membrane-associated complex I. Its components include TNFR1-associated death domain protein (TRADD), RIPK1, TNF receptor-associated factor 2 (TRAF2), cellular inhibitor of apoptosis proteins 1 and 2 (cIAP1/2), and the linear ubiquitin chain assembly complex (LUBAC) (12). Polyubiquitin chains on RIPK1 recruit transforming growth factor-β-activated kinase 1 (TAK1) and the IκB kinase (IKK) complex, activating nuclear factor-κB (NF-κB)-dependent pro-survival signaling (13). SPATA2–CYLD-mediated deubiquitination counterbalances this process (14, 15). In keratinocytes, inhibition of IKK/NF-κB signaling compromises this pro-survival checkpoint and can induce RIPK1-dependent necroptosis and skin inflammation (16).

When pro-survival signaling is impaired, RIPK1 leaves complex I and joins Fas-associated death domain protein (FADD) and caspase-8 in cytosolic complex II (17). Fully activated caspase-8 primarily drives apoptosis. Its heterodimer with the long isoform of cellular FLICE-like inhibitory protein (c-FLIP_L) instead suppresses necroptosis by cleaving RIPK1 and RIPK3 (18, 19). When caspase-8 is absent or inhibited, RIPK1 and RIPK3 interact through receptor-interacting protein homotypic interaction motifs (RHIMs), assembling the necrosome and activating RIPK3 (20). Activated RIPK3 phosphorylates MLKL, triggering its oligomerization and translocation to the plasma membrane. Membrane-associated MLKL oligomers then disrupt membrane integrity and execute necroptosis (21, 22).

### 3.2 Z-DNA-binding protein 1-driven cell death

Beyond canonical TNFR1 signaling, ZBP1 recognizes Z-form nucleic acids and recruits RIPK3 through RHIM-mediated interactions. In specific cellular or genetic contexts, this interaction can bypass RIPK1 and activate the RIPK3–MLKL execution axis (23). In keratinocytes, the RHIM-dependent scaffolding function of RIPK1 restrains ZBP1–RIPK3–MLKL signaling. Epidermis-specific *Ripk1* deletion or disruption of the RIPK1 RHIM releases this restraint, inducing keratinocyte necroptosis and IL-17-associated skin inflammation. Genetic ablation of *Zbp1*, *Ripk3*, or *Mlkl* ameliorates these phenotypes (23–25).

Nucleic acid sensing, autophagy, and ZBP1 splice isoforms further regulate this axis. In mice with keratinocyte-specific *Casp8* deletion, cytosolic DNA accumulation activates the cyclic GMP–AMP synthase (cGAS)–stimulator of interferon genes (STING) pathway, increases ZBP1 and MLKL expression, and promotes ZBP1-dependent necroptosis (26). Keratinocyte-specific *Atg9a* deletion similarly activates the TNF–cGAS–STING–type I interferon (IFN-I)–ZBP1 axis, with ZBP1-dependent apoptosis and necroptosis jointly driving severe dermatitis (27). Autophagy therefore acts as an important checkpoint restraining ZBP1-associated inflammatory cell death. In mice with epidermal *Ripk1* deletion, the short splice isoform ZBP1-S competitively binds Z-form nucleic acids and thereby limits ZBP1-mediated cell death and inflammation (28). Epidermal expression of constitutively active ZBP1 also induces FADD–caspase-8-dependent apoptosis in a manner requiring the RIPK1 RHIM but not RIPK1 kinase activity (29). Together, these findings indicate that ZBP1 can engage distinct cell-death programs depending on the genetic and signaling context.

### 3.3 Ubiquitin-dependent control of cell-death decisions

Ubiquitination further regulates cell-death decisions and necroptotic execution. LUBAC, composed of HOIP, HOIL-1, and SHARPIN, assembles linear (M1-linked) ubiquitin chains. SPATA2 recruits CYLD to the TNFR1 signaling complex, facilitating the removal of K63- and M1-linked chains, whereas OTULIN selectively hydrolyzes M1-linked chains (14, 30). Keratinocyte-specific loss of HOIP or HOIL-1 causes severe dermatitis driven mainly by TNFR1- and caspase-8-dependent apoptosis, independently of RIPK1 kinase activity. When TNFR1 is absent in these models, TRAIL and CD95L redundantly induce RIPK1 kinase-dependent apoptosis and necroptosis (31). SHARPIN-deficient dermatitis is likewise dominated by apoptosis, with only a minor necroptotic contribution (32). By contrast, keratinocyte-specific OTULIN deficiency induces TNFR1- and RIPK1 kinase-dependent cell death, with RIPK3–MLKL-mediated necroptosis as the predominant program. Nevertheless, FADD-dependent apoptosis still causes mild skin lesions when necroptosis is blocked (33).

Ubiquitination also directly regulates core necroptotic effectors. PELI1 recognizes RIPK3 phosphorylated at T182 and promotes its K48-linked polyubiquitination at K363, targeting RIPK3 for proteasomal degradation. In human neonatal epidermal keratinocytes, *PELI1* knockdown increased RIPK3 expression (34). K63-linked ubiquitination of MLKL at K219 facilitates higher-order assembly of phosphorylated MLKL (p-MLKL) at the plasma membrane and subsequent membrane rupture. Homozygous *Mlkl* K219R knock-in mice were resistant to experimentally induced necroptotic skin injury (35). Together, these findings indicate that ubiquitin homeostasis governs both cell-death pathway selection and necroptotic execution in keratinocytes, highlighting several potential intervention points within the ubiquitin-regulatory machinery.

## 4 Keratinocyte necroptosis in inflammatory skin diseases

### 4.1 Psoriasis

Keratinocyte necroptosis may contribute to psoriatic inflammation, but human evidence remains limited and inconsistent. One small histological study reported increased RIPK1 and MLKL throughout the lesional epidermis, with RIPK3 and p-MLKL concentrated in the upper layers (36). The p-MLKL signal supports pathway activation but does not establish MLKL-dependent necroptotic execution. By contrast, another study found reduced RIPK1 in lesional skin; in cultured human keratinocytes, RIPK1 downregulation also enhanced TRAIL-induced inflammatory mediator expression (37). This discrepancy may reflect clinical or methodological heterogeneity and the contrasting kinase and scaffolding functions of RIPK1. Total RIPK1 abundance is therefore an unreliable proxy for necroptotic activity (38). An early phase IIa trial reported exploratory target-plaque and histological improvements with the RIPK1 inhibitor GSK2982772 (39). A subsequent high-exposure study achieved peripheral target engagement and skin pharmacodynamic changes without meaningful clinical benefit (40). Neither trial directly assessed keratinocyte necroptosis.

Genetic evidence more strongly implicates RIPK3 while revealing functions beyond necroptotic execution. Whole-body *Ripk3* deletion attenuated imiquimod (IMQ)-induced inflammation; in corresponding keratinocyte experiments, *Ripk3* loss reduced inflammatory mediator expression without substantially altering cell death (41). Keratinocyte-specific *Ripk3* deletion also attenuated IMQ-induced dermatitis, while primary mouse keratinocytes showed RIPK3-dependent IL-36α/NF-κB signaling that persisted after *Mlkl* loss (42).

Pharmacological findings require cautious interpretation. Nec-1s and necrosulfonamide (NSA) suppressed necroptosis in HaCaT cells and attenuated IMQ-induced dermatitis (36). However, the NSA-reactive Cys86 residue in human MLKL is not conserved in mice, and NSA also targets gasdermin D (GSDMD) (43, 44). Saracatinib bound MLKL, inhibited its phosphorylation, oligomerization, and membrane translocation, and attenuated IMQ-induced dermatitis (45). Cabozantinib suppressed MLKL oligomerization and the release of damage-associated molecular patterns (DAMPs) and reduced IMQ-induced inflammation, although its effect on MLKL appeared indirect (46). Both mechanistic analyses used non-keratinocyte systems, and neither study established MLKL dependence of the observed *in vivo* protection. The quinazoline RIPK3 inhibitor compound 32 inhibited RIPK3 and MLKL phosphorylation and redirected cell death toward apoptosis *in vitro*; it also attenuated IMQ-induced dermatitis (47). Reducing oxidative stress similarly suppressed necroptosis-associated signaling in keratinocytes *in vitro* and alleviated IMQ-induced inflammation *in vivo* (48). By contrast, periplogenin and convallatoxin induced reactive oxygen species-dependent necroptosis in cultured keratinocytes, yet their topical application reduced epidermal hyperplasia and inflammation in mice with psoriasiform dermatitis (49, 50). Because these *in vivo* benefits were not shown to require keratinocyte necroptosis and may reflect other antiproliferative or anti-inflammatory effects, they do not establish a protective role for endogenous necroptosis.

Overall, the strongest causal evidence in psoriasis supports a proinflammatory role for keratinocyte RIPK3 that is at least partly independent of MLKL. Direct evidence for MLKL-dependent necroptotic execution in patient lesions and for therapeutic benefit from selectively targeting this process remains lacking. Target development should therefore account for the protective scaffolding function of RIPK1 and the non-death functions of RIPK3.

### 4.2 Dermatitis

#### 4.2.1 Interface dermatitis

Necroptotic signaling may contribute to epidermal injury in interface dermatitis dominated by type 1 immune responses. Lichen planus (LP) and cutaneous lupus erythematosus (CLE) lesions were enriched in IFN-γ- and TNF-α-producing T cells and showed increased epidermal RIPK3 expression (51). In primary human keratinocytes and three-dimensional epidermal models, IFN-γ/TNF-α or lesion-derived T-cell supernatants induced RIPK3 expression, MLKL phosphorylation, and cell death, whereas *RIPK3* knockdown reduced MLKL phosphorylation and death (51). These findings support RIPK3–MLKL-associated necroptotic signaling but do not establish MLKL-dependent execution. In pharmacologically sensitized reconstructed human epidermis, eclitasertib reduced T-cell supernatant-induced keratinocyte death and inflammatory mediator release while improving epidermal architecture. It also suppressed disease-associated gene signatures in ex vivo LP and CLE lesions (52). This provides human preclinical support for RIPK1 inhibition, although RIPK1 pleiotropy precludes necroptosis-specific attribution. Upstream tyrosine kinase 2 (TYK2) inhibition with deucravacitinib suppressed IFN-α-responsive genes in primary human keratinocytes, normalized epidermal alterations in reconstructed epidermis, and reduced *RIPK3* and *MLKL* transcripts in ex vivo CLE lesions (53). Clinical improvement was reported in four treatment-refractory patients receiving off-label deucravacitinib, but necroptosis was not assessed, precluding attribution of the benefit to inhibition of keratinocyte necroptosis (53).

Endogenous regulators may further shape the balance among cell-death programs. Expression of death-associated protein kinase 1 (DAPK1) was higher in LP lesions than in psoriatic or atopic dermatitis (AD) lesions. In primary human keratinocytes, IFN-γ/TNF-α induced DAPK1 expression, and IL-32 further enhanced this response. After stimulation with LP lesion-derived T-cell supernatants, *DAPK1* loss reduced apoptosis-associated protein levels and overall cell death while increasing RIPK3 expression and inflammatory gene expression (54). DAPK1 may therefore constrain RIPK3-associated inflammation by promoting apoptosis.

Cutaneous acute graft-versus-host disease (aGVHD) exhibits interface dermatitis-like histopathology and may involve a related IFN-γ-driven mechanism. Patient lesions showed increased epidermal ZBP1 expression and enhanced p-RIPK3 and p-MLKL signals (55). In primary human keratinocytes and human skin models, leukocyte-derived IFN-γ induced ZBP1 expression and cell death through JAK signaling, whereas JAK inhibition reduced cell death (55). These findings implicate IFN-γ–JAK–ZBP1 signaling in necroptosis-associated epidermal injury.

Overall, patient tissues and human models implicate necroptosis-related pathways in LP, CLE, and cutaneous aGVHD. Although upstream TYK2 inhibition has shown preliminary clinical activity in CLE, interventions directly targeting necroptosis remain preclinical. MLKL-dependent execution remains incompletely established, and the direct contribution of ZBP1 to LP and CLE remains unresolved.

#### 4.2.2 AD and contact hypersensitivity

AD is characterized by xerosis, recurrent eczema, and type 2 inflammation, with excessive colonization by *Staphylococcus aureus* as a prominent feature of cutaneous dysbiosis (56). In a lipid-depleted dry-skin model, *S. aureus* infection increased IL-33 release, group 2 innate lymphoid cell (ILC2) accumulation, and type 2 inflammation, whereas whole-body *Tlr2* deletion attenuated these phenotypes (57). In KERTr cells, infection increased RIPK3 and MLKL phosphorylation, whereas TLR2 blockade reduced cell injury and IL-33 release. Pharmacological blockade of RIPK1- or MLKL-associated signaling also reduced IL-33 release in KERTr cells and primary mouse keratinocytes (57). A separate study implicated TLR4/NLRP12-associated activation of multiple cell-death pathways in AD-like models. In stimulated HaCaT cells, *TLR4* or *NLRP12* overexpression increased cell death and p-MLKL alongside apoptotic and pyroptotic markers, whereas silencing either gene reduced cell death (58). These studies implicate necroptosis-related signaling in experimental AD-associated inflammation but do not establish MLKL-dependent necroptotic execution or a keratinocyte-specific causal contribution *in vivo*. The absence of evidence from patient lesions, together with limitations in pharmacological specificity and cross-species applicability, further constrains attribution to keratinocyte necroptosis in AD.

In an independent model of *S. aureus* skin infection, whole-body deletion of *Mlkl* increased bacterial burden and inflammation, whereas *Ripk3* deletion enhanced bacterial clearance and reduced inflammation. This divergence may reflect RIPK3 regulation of MLKL-independent processes, including IL-1β activation and apoptosis (59). MLKL may therefore support host defense during infection, but systemic knockout cannot identify the responsible cell type, attribute the phenotype to keratinocyte necroptosis, or justify direct extrapolation to AD.

By contrast, contact hypersensitivity provides stronger cell-specific evidence. Keratinocyte-specific *Mlkl* deletion reduced skin lesions, cell death, high mobility group box 1 (HMGB1) release, and neutrophil activation, supporting MLKL-dependent keratinocyte death as an amplifier of local inflammation (60). Overall, current evidence does not support a uniformly proinflammatory role for keratinocyte necroptosis across different forms of dermatitis; its biological and therapeutic relevance should instead be evaluated in relation to disease context and infection status.

## 5 Keratinocyte necroptosis in acute epidermal injury

Within the broader spectrum of acute epidermal injury, this section focuses on UVB-induced injury and SJS/TEN, which represent environmentally induced and drug-specific immune-mediated injury, respectively.

### 5.1 UVB-induced injury

UVB radiation is a major extrinsic cause of cutaneous photodamage. In HaCaT cells, UVB reduced RIPK1 expression and activated NF-κB while inducing RIPK3 and MLKL phosphorylation independently of RIPK1 kinase activity. RIPK3 inhibition or *RIPK3* knockdown reduced MLKL phosphorylation, cell death, and inflammatory mediator release; whole-body *Ripk3* deletion likewise attenuated epidermal p-MLKL, cell death, and skin injury in mice (61). However, the systemic model does not establish the responsible cell type, and RIPK3 also contributes to mitochondrial apoptosis (61). UVB also upregulated TET2; reducing TET2 expression suppressed RIPK3–MLKL signaling and cell death in cultured keratinocytes and attenuated photodamage *in vivo* (62). RIPK1 responses varied across experimental models. A separate study reported UVB-induced RIPK1 upregulation and NF-κB activation in HaCaT cells, human dermal fibroblasts, and mouse skin (63). In human dermal fibroblasts, RIPK3 and MLKL exerted necroptosis-independent pro-survival and pro-apoptotic effects, respectively (64). These discrepancies suggest that the regulation and functions of these molecules depend on UVB dose, observation time, and cellular context.

UVB-induced keratinocyte death showed substantial redundancy among cell-death pathways. Keratinocyte-specific loss of either *Gsdmd* or *Mlkl* did not reduce overall cell death, whereas whole-body *Zbp1* deletion suppressed pyroptosis-, apoptosis-, and necroptosis-related signaling and reduced epidermal cell death and thickening without substantially reducing inflammatory-cell infiltration (65). Thus, RIPK3–MLKL-associated necroptosis is one component of UVB-induced epidermal injury rather than its sole cell-death mechanism. ZBP1-associated activation of multiple death pathways and the phenotypic dissociation between epidermal injury and immune infiltration suggest that blocking a single pathway may provide insufficient anti-inflammatory benefit.

### 5.2 SJS/TEN

Unlike UVB-induced environmental stress, SJS/TEN is driven primarily by drug-specific immune responses and is characterized by widespread keratinocyte death and epidermal detachment. TEN lesions showed increased RIPK3 expression and p-MLKL signals predominantly in epidermal keratinocytes, suggesting RIPK3–MLKL pathway activation (66). In HaCaT cells and primary human neonatal epidermal keratinocytes, *RIPK3* overexpression increased MLKL phosphorylation and cell death, whereas *MLKL* knockdown reduced cell death. Dabrafenib likewise reduced these responses (66), although its nonselectivity precludes attribution solely to RIPK3 inhibition.

Supernatants from culprit-drug-stimulated peripheral blood mononuclear cells of patients with SJS/TEN induced RIPK1- and RIPK3-dependent cell death in patient-derived keratinocytes through annexin A1 binding to formyl peptide receptor 1 (FPR1) (67). In the presence of antigen-presenting cells, culprit drug-activated CD8^+^ T cells produced lipocalin-2, which induced neutrophil extracellular trap (NET) formation. NET-derived LL-37 upregulated FPR1 in keratinocytes, increasing their susceptibility to annexin A1-induced death. Damaged keratinocytes released additional LL-37, forming a positive feedback loop that amplified epidermal injury and inflammation (68).

Overall, SJS/TEN-associated keratinocyte necroptosis may represent an epidermal effector process driven jointly by drug-specific T-cell responses and neutrophil activation and amplified through annexin A1–FPR1 signaling, rather than an isolated cell-intrinsic event. However, the dependence of this immune–epidermal loop on MLKL-mediated execution and its amenability to therapeutic intervention remain to be established. The molecular regulation and context-dependent roles of keratinocyte necroptosis across the disease and injury contexts reviewed here are summarized in **Figure 1**.

**Figure 1:**
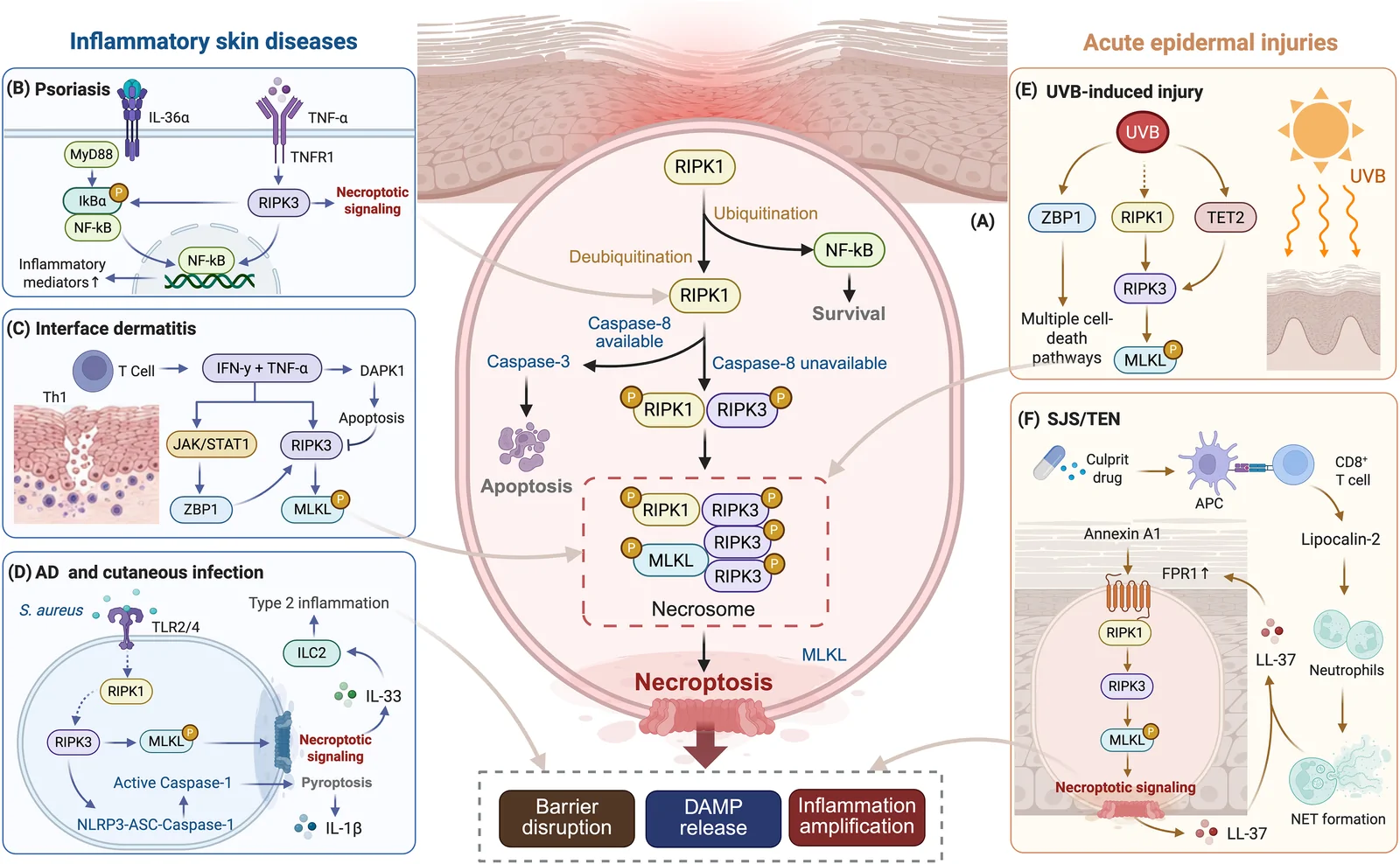
Molecular regulation and context-dependent roles of keratinocyte necroptosis in inflammatory skin diseases and acute epidermal injuries. Panel **(A)** illustrates the core molecular machinery governing keratinocyte necroptosis, whereas panels **(B–F)** summarize its context-dependent roles across the disease and injury settings reviewed. In these contexts, keratinocyte necroptosis may disrupt the epidermal barrier, promote DAMP release, and amplify inflammation (Created with BioRender.com).

## 6 Conclusions and future perspectives

Keratinocyte necroptosis is a context-dependent hub linking epidermal barrier disruption, immune activation, and tissue injury. Its biological effects are shaped by initiating stimuli, infection status, and the immune microenvironment. They also depend on the balance between the scaffolding and kinase functions of RIPK1, the cell-death-independent functions of RIPK3 and MLKL, and transitions among cell-death programs. Candidate intervention strategies include inhibition of RIPK1 or RIPK3, blockade of MLKL-dependent necroptotic execution, and modulation of ZBP1-mediated nucleic acid sensing or ubiquitin homeostasis. However, necroptosis-related signaling in keratinocytes should not be viewed as a linear pathway governed by a single molecular node. Limited drug selectivity, protein pleiotropy, and compensatory switching among death programs mean that inhibiting an individual pathway component cannot be equated with selectively blocking pathogenic keratinocyte necroptosis. Systemic pathway inhibition may compromise host defense against infection. Consequently, protective effects of pathway modulation in animal models may not translate into clinical benefit. Using an author-derived qualitative framework, **Table 1** summarizes disease- and injury-specific evidence, the extent of support for MLKL-dependent necroptotic execution, and key translational limitations.

**Table 1:** Disease- and injury-specific evidence and translational implications of keratinocyte necroptosis.

r	Trigger and core signaling axis	Key evidence	Evidence for MLKL-dependent necroptotic execution	Pathobiological role and intervention outcomes	Evidence strength and key limitations
Psoriasis/IMQ-induced psoriasiform dermatitis (42)	IMQ/IL-36α → RIPK3-dependent NF-κB signaling in keratinocytes, partly independent of MLKL.	• Patient lesions showed increased epidermal RIPK3. • Keratinocyte-specific *Ripk3* deletion attenuated IMQ dermatitis; primary mouse keratinocytes showed RIPK3-dependent NF-κB activation that persisted after *Mlkl* loss.	Limited: RIPK3 causality in keratinocytes was established, but MLKL-dependent cell death or tissue injury was not directly demonstrated.	Keratinocyte RIPK3 promoted psoriasiform inflammation partly through MLKL-independent IL-36α–NF-κB signaling; its deletion attenuated the IMQ phenotype.	Limited for MLKL-dependent execution; keratinocyte-specific evidence establishes RIPK3 causality, whereas human histology is associative and the evidence remains predominantly preclinical.
Psoriasis/IMQ-induced psoriasiform dermatitis (45, 46)	TNF-induced necroptosis → MLKL phosphorylation, oligomerization, and membrane translocation.	• Saracatinib bound MLKL and inhibited its activation and membrane execution. • Cabozantinib inhibited MLKL oligomerization and DAMP release.	Supported in non-keratinocyte cells; MLKL dependence was not established in keratinocytes or *in vivo*.	Both agents attenuated inflammation and epidermal hyperplasia in IMQ-treated mice.	Limited for keratinocyte MLKL-dependent execution; mechanistic assays used non-keratinocyte cells, and multitarget or indirect drug actions limit attribution of the *in vivo* benefit to MLKL.
Chronic plaque psoriasis (40)	High-exposure RIPK1 kinase inhibition with modified-release GSK2982772.	A randomized, placebo-controlled study (n=29; 18 completed) achieved near-complete peripheral target engagement and skin pharmacodynamic changes.	Not directly assessed: necroptosis markers and MLKL-dependent execution were not evaluated.	No meaningful clinical benefit was observed; PASI75 response was not superior to placebo.	Not directly assessed for MLKL-dependent execution; the randomized trial was limited by its small sample and attrition and did not evaluate keratinocyte necroptosis.
Interface dermatitis: LP/CLE (51, 52)	IFN-γ/TNF-α or lesion-derived T-cell supernatants → RIPK3–MLKL-associated keratinocyte death and RIPK1-inhibitor-sensitive epidermal injury.	• Patient lesions showed increased epidermal RIPK3. • In primary human keratinocytes, *RIPK3* knockdown reduced p-MLKL and cell death. • Eclitasertib reduced cell death in sensitized reconstructed human epidermis and suppressed inflammatory programs in ex vivo lesions.	*RIPK3* knockdown reduced p-MLKL and cell death, but MLKL loss-of-function and membrane execution were not tested.	Type 1 immunity promoted epidermal injury; RIPK1 inhibition showed human-derived preclinical activity.	Moderate for MLKL-dependent execution; the human-derived models lacked MLKL loss-of-function testing, while experimental sensitization and RIPK1 pleiotropy limit attribution. Clinical efficacy remains untested.
Cutaneous aGVHD (55)	Leukocyte-derived IFN-γ → JAK/STAT1–ZBP1–RIPK3–MLKL signaling.	• Patient lesions showed epidermal ZBP1, p-RIPK3 and p-MLKL. • Primary human keratinocytes and human skin models demonstrated IFN-γ/JAK-dependent ZBP1 induction and cell death; JAK inhibition was protective.	p-MLKL membrane localization and RIPK3-inhibitor-sensitive death were observed, but functional MLKL dependence was not tested.	Links type 1 immunity to ZBP1-associated epidermal death; JAK inhibition protected human-derived models.	Moderate for MLKL-dependent execution; human-derived evidence lacked functional MLKL testing, while JAK inhibition was upstream and not necroptosis-selective. Clinical efficacy was not assessed.
AD-associated dry-skin inflammation/cutaneous *S. aureus* infection (57, 59)	*S. aureus* → TLR2-associated RIPK3/MLKL signaling and IL-33 release.	• KERTr cells showed RIPK3/MLKL phosphorylation; pathway inhibitors reduced IL-33 release in KERTr cells and primary mouse keratinocytes. • Systemic *Mlkl* loss worsened infection, whereas *Ripk3* loss improved bacterial clearance.	Not established in AD; keratinocyte-specific *Ripk3*/*Mlkl* validation was lacking.	RIPK3–MLKL-associated signaling may amplify type 2 inflammation, whereas MLKL may support host defense during infection.	Limited for keratinocyte MLKL-dependent execution; evidence relies on phosphorylation, inhibitors, and systemic knockout without AD patient-tissue or cell-specific validation.
Contact hypersensitivity (60)	DNCB → RIPK1–RIPK3–MLKL signaling → HMGB1 release and inflammatory cell recruitment.	• Keratinocyte-specific *Mlkl* deletion reduced dermatitis, cell death, HMGB1 release, and inflammatory cell recruitment. • Nec-1 and topical TC13172 also attenuated dermatitis.	Strong functional evidence from keratinocyte-specific *Mlkl* loss; membrane execution was not directly assessed.	MLKL-dependent death amplified HMGB1-mediated inflammation; genetic and pharmacological inhibition attenuated dermatitis.	Strong for functional MLKL dependence in murine keratinocytes; membrane execution was not directly assessed, human validation is lacking, and pharmacological selectivity remains limited.
UVB-induced epidermal injury (61, 65)	UVB → RIPK3–MLKL activation and ZBP1-associated multi-pathway cell death.	• Human sun-exposed skin showed increased p-MLKL alongside apoptotic and pyroptotic markers. • In HaCaT cells, RIPK3 inhibition or *RIPK3* knockdown reduced p-MLKL and cell death. • Keratinocyte-specific *Mlkl* loss did not reduce total cell death, whereas systemic *Ripk3* or *Zbp1* loss attenuated UVB injury.	Limited: p-MLKL indicated pathway activation, but keratinocyte-specific *Mlkl* loss did not reduce total cell death.	RIPK3 promoted UVB injury; *Zbp1* loss reduced cell death and epidermal thickening, but not immune-cell infiltration.	Limited for MLKL-dependent execution; human findings are associative, keratinocyte-specific *Mlkl* loss did not reduce total cell death, and systemic knockouts and overlapping death programs limit attribution.
SJS/TEN (66)	RIPK3 upregulation under SNP stress → MLKL phosphorylation and membrane localization.	• TEN lesions showed increased epidermal RIPK3 and p-MLKL. • In HaCaT/HEKn cells, *RIPK3* overexpression enhanced p-MLKL and cell death, whereas *MLKL* knockdown reduced death.	Supported *in vitro* by p-MLKL membrane localization and reduced cell death following *MLKL* knockdown.	RIPK3 sensitized keratinocytes to MLKL-dependent death; dabrafenib reduced p-MLKL and cell death *in vitro*.	Strong *in vitro* for MLKL-dependent execution in human keratinocyte models; patient evidence remains histological, while artificial overexpression/SNP stress and nonselective dabrafenib limit translation.
SJS/TEN (67)	Culprit-drug-stimulated PBMC supernatant → annexin A1–FPR1 → RIPK1/RIPK3-dependent keratinocyte death.	• In patient-derived PBMC/keratinocyte models, annexin A1 depletion, FPR1 blockade, or *RIPK3* knockdown reduced cell death. • NSA was protective *in vitro* and in the mouse model.	Not established: p-MLKL and genetic MLKL dependence were not assessed; NSA alone was insufficient to prove MLKL-dependent execution.	Annexin A1–FPR1 signaling linked drug-specific immune activation to keratinocyte death; pathway blockade protected experimental models.	Limited for MLKL-dependent execution; patient-derived evidence supports the upstream annexin A1–FPR1 axis, but MLKL dependence was not tested. NSA selectivity/species applicability and unresolved *in vivo* cell sources further limit attribution.

The evidence-strength categories were developed by the authors as a qualitative framework for this review and do not constitute a validated evidence-grading system. They indicate how strongly the available evidence supports MLKL-dependent necroptotic execution in keratinocytes, rather than the overall quality of individual studies. “Strong” denotes keratinocyte-specific genetic evidence establishing functional dependence on MLKL or convergent human-derived evidence combining MLKL loss-of-function with execution-stage readouts. “Moderate” denotes human-derived or causal pathway evidence without complete validation of MLKL-dependent execution. “Limited” denotes expression or phosphorylation changes, pharmacological evidence, or systemic models lacking cell-specific attribution. Tissue p-MLKL supports pathway activation but does not by itself establish MLKL oligomerization, membrane translocation, or lytic cell death. Human lesional staining provides localization or association evidence only, whereas systemic knockout does not establish keratinocyte autonomy.

IL-36α, interleukin-36 alpha; PASI75, ≥75% improvement from baseline in the Psoriasis Area and Severity Index; IFN-γ, interferon gamma; JAK, Janus kinase; STAT1, signal transducer and activator of transcription 1; p-RIPK3, phosphorylated receptor-interacting protein kinase 3; TLR2, Toll-like receptor 2; IL-33, interleukin-33; KERTr, human keratinocyte cell line; DNCB, 2,4-dinitrochlorobenzene; Nec-1, necrostatin-1; HaCaT, spontaneously immortalized human keratinocyte cell line; SNP, sodium nitroprusside; HEKn, neonatal human epidermal keratinocytes; PBMC, peripheral blood mononuclear cells.

Future studies should prioritize local drug delivery and stratify patients on the basis of disease subtype, infection status, and composite biomarker panels that incorporate, rather than rely solely on, p-MLKL. Orthogonal validation combining keratinocyte-specific genetics with pharmacology should determine whether the pathogenic effects are keratinocyte-autonomous, identify the relevant molecular function, and define the therapeutic window. Clinical studies should evaluate target engagement or pathway inhibition, changes in inflammatory activity, and clinical outcomes as distinct endpoints. They should also distinguish changes in the abundance of pathway proteins and cell-death-independent signaling from MLKL-dependent necroptotic execution in keratinocytes. At present, evidence from patient tissues and human-derived models is most developed for SJS/TEN, LP, and CLE. Histological findings in psoriasis are inconsistent, and early clinical trials of RIPK1 inhibitors have not demonstrated clear efficacy. Evidence in AD, by contrast, remains predominantly derived from experimental models rather than patient studies. Overall, strategies targeting keratinocyte necroptosis remain largely preclinical, with only limited early clinical evaluation. Their appropriate disease indications, target populations, and capacity to deliver meaningful clinical benefit remain to be defined.
